# Safety-netting communication during telephone consultations: an observational study using recorded consultations

**DOI:** 10.3399/BJGP.2025.0637

**Published:** 2026-06-02

**Authors:** Peter J Edwards, Barbara Caddick, Adam R Skeen, Jordan Lin, Helena Thornton, Matthew J Ridd, Rebecca K Barnes, Chris Salisbury

**Affiliations:** 1 Centre for Academic Primary Care, Bristol Medical School, University of Bristol, Bristol, UK; 2 University of Birmingham, Birmingham, UK; 3 Somerset NHS Foundation Trust, Somerset, UK; 4 Nuffield Department of Primary Care Health Sciences, University of Oxford, Oxford, UK

**Keywords:** Remote consulting, primary health care, general practice, quality assurance, safety-netting, patient safety

## Abstract

**Background:**

In 2024, one-third of NHS GP consultations in England were conducted by telephone. Although remote consulting can be convenient for patients and GPs, it may increase diagnostic uncertainty. Safety-netting advice (guidance on when, and how, patients should seek further medical help) is a tool used to mitigate clinical risk, but its delivery during telephone consultations has not been studied in detail.

**Aim:**

To evaluate the communication, documentation, and patient recall of safety-netting advice in GP telephone consultations.

**Design & setting:**

Observational study using data from seven GP practices in south west England.

**Method:**

Practices routinely recording telephone consultations were invited to participate. Patients with a recent telephone consultation with a participating clinician were invited to consent to consultation recording retrieval, medical record extraction, and completion of a post-consultation questionnaire. Recordings of 96 telephone consultations were obtained and coded using the validated Safety-Netting Coding Tool. Regression models explored factors associated with safety-netting advice. Patient recall was assessed using post-consultation questionnaires.

**Results:**

There were 93 instances of safety-netting advice, delivered in 60.4% (*n* = 58) of 96 consultations applying to 43.4% (*n* = 72) of 166 identified problems. Instances of safety-netting advice were mostly GP initiated (95.7%, *n* = 89), delivered during treatment planning (66.7%, *n* = 62), and included specific elements (64.5%, *n* = 60). Delivered safety-netting advice was documented in 64.2% (*n* = 34) of 53 consultations with notes that were available. Written advice was rarely used: only four out of 96 consultations contained written advice, which was delivered via text message. Patients correctly recalled the presence of safety-netting advice in two-thirds of consultations when it was given. Safety-netting advice was more likely to be provided by younger GPs (aged <45 years; odds ratio 5.09, *P* = 0.011).

**Conclusion:**

Safety-netting advice was commonly delivered during GP telephone consultations, but its delivery, documentation, and recall were inconsistent. Opportunities exist to improve the consistency, documentation, and use of written advice to support patient understanding, recall, and safety in remote care.

## How this fits in

Safety-netting advice is a key strategy to manage diagnostic uncertainty, but prior studies have not examined its delivery during telephone consultations in detail. In this study, GPs gave safety-netting advice in 60.4% of telephone consultations — a rate comparable to that of face-to-face consultations — but, often, this was done only verbally, with inconsistent documentation. Safety-netting advice was more likely to be delivered by younger GPs, and patients’ variable recall of advice indicated potential gaps in communication. These findings suggest opportunities exist to improve the consistency and communication of safety-netting through digital innovations, such as artificial intelligence scribes or prompts, which could generate written advice for patients and prompt GPs when safety-netting advice is omitted.

## Introduction

In 2024, one-third of GP appointments in England were conducted by telephone, a figure comparable to pre-COVID-19 pandemic levels in 2019.^1–3^ This represents a marked shift from the ‘remote by default’ model that was adopted during the COVID-19 pandemic in England.^2^ When used appropriately, telephone consulting can improve access, support continuity, and provide an efficient way to deliver health care.^4^ It may also help lower health care’s carbon footprint.^5^ However, telephone consultations lack visual cues — a key element of clinical assessment — and they are typically shorter, cover fewer problems, involve less information gathering and advice giving, and allow for less rapport building than face-to-face assessments.^6,7^ Consequently, telephone consulting is often perceived as higher risk — particularly for undifferentiated problems — and may increase diagnostic uncertainty.^2,8,9^

Safety-netting is a key strategy to manage diagnostic uncertainty, particularly in remote consulting.^9–11^ Roger Neighbour first described this as an in-consultation reflective tool, whereby clinicians considered ‘what if?’ scenarios, including the possibility of missed or incorrect diagnoses.^12^ The term has since been applied to a range of clinical activities, but ‘safety-netting advice’ is synonymous with Neighbour’s original description and defined as: *‘Information shared with a patient or their carer* [that is] *designed to help them identify the need to seek further medical help if their condition fails to improve, changes, or if they have concerns about their health.’* ^13^

Previous studies of recorded consultations have examined safety-netting communication during face-to-face GP consultations^14,15^ and physiotherapy consultations.^16^ Analyses of recorded telephone consultations have reported only whether safety-netting was present or absent,^17,18^ but none have explored in detail how it is communicated. This study, therefore, aimed to:

- examine when, and how, GPs communicated safety-netting advice in telephone consultations;
- explore GPs’ views on its utility; and
- assess patients’ recall.

Findings were then compared with those of face-to-face consultations to identify opportunities to improve practice.

## Method

### Data

Consultations were obtained from the Telesafe research archive, full details of which have been reported elsewhere.^19^ Briefly, GP practices in south west England that routinely recorded all telephone consultations were recruited and their healthcare professionals (HCPs) were invited to participate. In total, 28 HCPs from seven practices consented and allowed the research team to access their recorded consultations with patients who had also consented to participate. HCP participants completed a pre-study electronic questionnaire (see Supplementary Information S1) so that their demographics and views on safety-netting advice could be collected.

Patients were invited by participating practices by post or text message if they had had a telephone consultation within the previous 14 days with a participating clinician and met the eligibility criteria, which included being an adult (aged ≥18 years) who was consulting for themselves. Patients were excluded if they were receiving end-of-life care, lacked capacity to consent, or were unable to consult in English. Patients were asked to:

- consent to the retrieval of their consultation recording and medical records dating from 1 month before their consultation to 3 months after it; and
- complete a post-consultation survey (see Supplementary Information S2) online or on paper.

Data were collected on consultations that took place between April 2023 and June 2024.

### Coding

The content of consultations, including the number of problems discussed, was coded by four researchers using the Complex Consultations toolkit.^20^ Problem types were coded using the International Classification of Primary Care (ICPC) (version 3)^21^ and checked by the first author. The audio files of all consultations were screened for safety-netting advice by two coders. Safety-netting episodes were coded by the first author, using the validated Safety-Netting Coding Tool (SaNCoT).^13^ Inter-rater reliability of SaNCoT coding by this research team has been reported elsewhere.^13,22,23^

Notably, SaNCoT differentiates between:

- safety-netting advice (conditional follow-up, whereby a symptom or condition must occur for further help seeking to take place); and
- unconditional follow-up.

Follow-up included planned appointments with an HCP for that problem and related investigations (for example, blood tests).

SaNCoT also distinguishes between:

- generic advice, such as: ‘if it gets worse or doesn’t improve, please come back*’*; and
- specific advice, which names a new symptom/conditional (for example, haemoptysis, chest pain, and spreading redness) or specifies a timeframe (for example: ‘if it’s not better in 2 weeks, please come back*’*).

Minor adjustments were made for this study (see Supplementary Information S3); as an example, the patient response code ‘nods only’ was excluded because it is not applicable to telephone consultations.

### Software and statistical analysis

SaNCoT codes were transferred from Microsoft Excel to Stata/MP (version 19.5) for analysis; univariable and multivariable logistic regression models were applied to generate odds ratios (ORs) and 95% confidence intervals (CIs). Mixed-effects models accounted for the clustering of the following: problems within the same consultation, consultations by the same GP, and GPs at the same practice. The Stata code used to generate results is available online.^3^ Jupyter Notebook using Python was used to concatenate data from the per-problem level to the per-consultation level.

## Results

### Patient and consultation characteristics

Patients who consented to participate consulted with one of 20 participating GPs. There were slightly more female GPs (*n* = 12, 60.0%) than male GPs, most GPs were partners (*n* = 14, 70.0%), and most reported their ethnic group as White (*n* = 17, 85.0%) (Table 1). Each GP contributed a mean of 4.8 consultations (standard deviation [SD] 5.6, median = 2.5, range = 1–20) (data not shown). More consultations came from practices in the least deprived areas (65.6%, Index of Multiple Deprivation [IMD] deciles 9–10) than the most deprived areas (28.1%, IMD deciles 1–2) (Table 1).

**Table 1.** GP, patient, and consultation characteristics

Characteristic	***n***	**%**
**Patients,** ***n*** **= 93**		
Gender		
Male	34 (35)^a^	36.6
Female	59 (61)^a^	63.4
Age, years		
18–34	9	9.7
35–49	9	9.7
50–64	21 (22)^a^	22.6
≥65	53 (55)^a^	57.0
Not reported	1	1.1
Ethnic group		
White	87 (90)^a^	93.5
Other	3	3.2
Not reported/prefer not to say	3	3.2
**GPs,** ***n*** **= 20**		
Gender		
Male	8	40.0
Female	12	60.0
Age, years		
18–34	2	10.0
35–49	13	65.0
50–64	5	25.0
≥65	0	0.0
Not reported	0	0.0
Ethnic group		
White	17	85.0
Other	3	15.0
Not reported/prefer not to say	0	0.0
Role		
Partner	14	70.0
Salaried GP	5	25.0
GP registrar	1	5.0
**Consultations, *n* = 96**		
Consulting practitioner		
Partner	86	89.6
Salaried GP	9	9.4
GP registrar	1	1.0
Number of problems per consultation		
1	53	55.2
2	22	22.9
3	17	17.7
≥4	4	4.2
Consultations per practice IMD decile		
1 (most deprived)	20	20.8
2	7	7.3
5	6	6.3
9	42	43.8
10 (least deprived)	21	21.9

^a^Three patients had two separate telephone consultations. Data in brackets factor in double counting for the patients who had two consultations each. IMD = Index of Multiple Deprivation.

In total, 123 patients consented to join the study but only 101 consultation recordings were retrieved by practices. Five recordings were unusable (one answerphone message, one cut out mid-consultation, two with substantial third-party contributions, and one arranged a call-back but no further recording was sent to the research team). In total, 96 telephone consultations with 93 unique patients were included; three patients had two consultations each. Most patients were White (93.5%) and female (63.4%) (Table 1), and the mean patient age was 63.7 years (SD 16.4, median = 69, range = 18–94). Consultations involved a mean of 1.7 problems (SD 1.0, median = 1, range = 1–5) (data not shown), with 44.8% classified as multiproblem (at least two problems) (Table 1).

### Safety-netting per consultation

Safety-netting advice was provided in 60.4% (*n* = 58) of 96 consultations, while 77.1% (*n* = 74) included some form of follow-up. In five consultations without safety-netting advice, the GP planned a same-day face-to-face review. Almost all consultations (*n* = 92) included either safety-netting or follow-up, and 41.7% (*n* = 40) had both (data not shown).

### Safety-netting per problem

Table 2 outlines data on safety-netting advice and follow-up by problem type. Most of the 166 problems had either safety-netting advice or follow-up (83.7%, *n* = 139). In total, 24.1% (*n* = 40) of problems had both; however, clinicians more often arranged follow-up (64.5%, *n* = 107) than provided safety-netting (43.4%, *n* = 72).

**Table 2.** Safety-netting and follow-up by type of problem raised

Problem type (ICPC-3)	Problems, *n* (% of all 166 problems)	Follow-up present, *n* (%)	Safety-netting advice present, *n* (%)	Safety-netting advice and/or follow-up present, *n* (%)
A – General	31 (18.7)	20 (64.5)	13 (41.9)	25 (80.6)
B - Blood, blood-forming organs, and immune system	4 (2.4)	3 (75.0)	2 (50.0)	4 (100)
D - Digestive system	17 (10.2)	9 (52.9)	4 (23.5)	11 (64.7)
F - Eye	2 (1.2)	0 (0.0)	1 (50.0)	1 (50.0)
G - Genital system	17 (10.2)	13 (76.5)	8 (47.1)	16 (94.1)
H - Ear	2 (1.2)	0 (0.0)	1 (50.0)	1 (50.0)
K - Circulatory system	16 (9.6)	14 (87.5)	9 (56.3)	15 (93.8)
L - Musculoskeletal system	19 (11.4)	15 (78.9)	9 (47.4)	18 (94.7)
N - Neurological system	6 (3.6)	4 (66.7)	3 (50.0)	6 (100)
P - Psychological, mental, and neurodevelopmental	7 (4.2)	3 (42.9)	4 (57.1)	6 (85.7)
R - Respiratory system	7 (4.2)	3 (42.9)	5 (71.4)	6 (85.7)
S - Skin	13 (7.8)	7 (53.8)	5 (38.5)	10 (76.9)
T - Endocrine, metabolic, and nutritional system	13 (7.8)	9 (69.2)	4 (30.8)	11 (84.6)
U - Urinary system	9 (5.4)	7 (77.8)	3 (33.3)	8 (88.9)
W - Pregnancy and childbearing	2 (1.2)	0 (0.0)	1 (50.0)	1 (50.0)
Z - Social problems	1 (0.6)	0 (0.0)	0 (0.0)	0 (0.0)
Total	166 (100.0)	107 (64.5)	72 (43.4)	139 (83.7)

ICPC-3 = International Classification of Primary Care, third edition.

### Factors associated with safety-netting advice

In univariable modelling, safety-netting advice was less likely when problems were assessed later in the consultation (OR 0.54 per unit increase, 95% CI = 0.33 to 0.91, *P* = 0.020) and more likely when assessed by GPs aged <45 years (OR 3.56, 95% CI = 1.11 to 11.39, *P* = 0.033). A weak association was seen for problem acuity, with higher odds for acute rather than chronic problems (OR 2.67, 95% CI = 0.95 to 7.55, *P* = 0.063) (Table 3).

**Table 3.** Factors associated with safety-netting advice^a^

Variable category		Univariable modelling(*n* = 166 problems)					Multivariable modelling(*n* = 165 problems)		
	Covariate	*n*	Safety-netting advice, %	OR	95% **CI**	*P-value*	OR	95% **CI**	*P*-value
**Problem**	**Acuity**								
	Chronic	82	37.8	1	—	—	1	—	—
	Acute (includes acute flare of chronic problem)	84	48.8	2.67	0.95 to 7.55	0.063	1.69	0.57 to 5.02	0.344
	**Presentation**								
	Not first presentation	127	44.1	1	—	—	1	—	—
	First presentation	29	44.8	1.46	0.45 to 4.77	0.527	1.18	0.34 to 4.07	0.792
	Unclear	10	30.0	0.38	0.06 to 2.45	0.307	0.38	0.05 to 2.80	0.345
	**Order assessed by GP**								
	1	96	51.0	—	—	—	—	—	—
	2	43	39.5	—	—	—	—	—	—
	≥3	27	22.2	—	—	—	—	—	—
	Per unit increase	—	—	0.54	0.33 to 0.91	0.020	0.60	0.35 to 1.04	0.067
	**Follow-up**								
	No follow-up discussed	59	54.2	1	—	—	1	—	—
	Follow-up discussed	107	37.4	0.49	0.21 to 1.14	0.097	0.62	0.25 to 1.55	0.306
**GP**	**GP age, years**								
	≥45	91	35.2	1	—	—	1	—	—
	<45	75	53.3	3.56	1.11 to 11.39	0.033	5.09	1.45 to 17.89	0.011
**Patient**	**Age, years**								
	<65	71	45.1	1	—	—	1	—	—
	≥65	94	41.5	0.63	0.21 to 1.88	0.405	0.98	0.33 to 2.93	0.975
	**Gender**								
	Male	56	39.3	1	—	—	1	—	—
	Female	110	45.5	1.60	0.55 to 4.66	0.389	1.39	0.47 to 4.11	0.550
**Practice**	**Deprivation status**								
	Least deprived (IMD deciles 9–10)	100	40.0	1	—	—	1	—	—
	Middle (IMD decile 5)	9	66.7	4.62	0.49 to 43.5	0.181	9.5	0.86 to 105.4	0.067
	Most deprived (IMD deciles 1–2)	57	45.6	1.46	0.43 to 4.95	0.541	3.37	0.93 to 12.25	0.065

a Multivariable model and univariable age model excluded one patient for whom age was not available. IMD = Index of Multiple Deprivation. OR = odds ratio.

In multivariable modelling, GP age remained significant (OR 5.09, 95% CI = 1.45 to 17.89, *P* = 0.011), while problem order was weaker (OR 0.60 per unit increase, 95% CI = 0.35 to 1.04, *P* = 0.067). A weak association was also observed for practice deprivation level when comparing practices that were least deprived (IMD deciles 9–10) with those that were most deprived (IMD deciles 1–2) (OR 3.37, 95% CI = 0.93 to 12.25, *P* = 0.065). No statistically significant associations were found with first presentation, follow-up, patient age, or gender (Table 3).

### Content of safety-netting advice

Across 58 consultations that featured safety-netting advice, 93 instances of such advice were observed (Table 4). Most were GP initiated (95.7%, *n* = 89). Most advice contained specific elements (64.5%, *n* = 60), and advice was delivered during the closing stage of consultations (20.4%, *n* = 19) or in the diagnosis (8.6%, *n* = 8) and treatment planning (66.7%, *n* = 62) stages. Advice given during the closing stages was usually generic (68.4%, *n* = 13/19), whereas that given in the diagnosis and treatment stages was mainly specific (100.0% [*n* = 8/8] and 69.4% [*n* = 43/62], respectively) (data not shown). Patients were usually advised to seek further help at their GP surgery (77.4%, *n* = 72/93 instances of safety-netting advice), either by returning to the practice (69.4%, *n* = 50) or the same GP (30.6%, *n* = 22) (Table 4). In a few instances patients were told to call NHS 111 (2.2%, *n* = 2) or 999 (7.5%, *n* = 7).

**Table 4.** Content of safety-netting advice across all instances (*N* = 93)

Question	Code	*n*	**%**
Applicable to problem, treatment/management, or both	Problem	62	66.7
	Treatment/management	17	18.3
	Both	14	15.1
Consultation stage	Establishing reason for consultation	0	0.0
	Gathering information	2	2.2
	Delivering diagnosis	8	8.6
	Treatment planning	62	66.7
	Closing	19	20.4
	Post-consultation (text or call back)	2	2.2
Initiation	Patient	4	4.3
	Clinician	89	95.7
Format	Conditional plus course of action (for example, ‘if X happens, do Y action’)	87	93.5
	Conditional warning only (for example, ‘I would only be worried if X happened’)	6	6.5
Strength of endorsement	Weak (for example, can and could)	24	25.8
	Neutral	47	50.5
	Strong (for example, must and should)	22	23.7
Number of conditionals/symptoms to look out for (for example, worsening pain, symptoms persist, and new weakness)	1	60	64.5
	2	17	18.3
	3	5	5.4
	4	1	1.1
	≥5	10	10.8
Generic or specific advice	Generic (for example, problems, issues, concerns, and worse)	33	35.5
	Specific (for example, cough up blood and chest pain)	45	48.4
	Both	15	16.1
Action advised	No action (conditional warning only)	6	6.5
	Contact other in-hours medical service	2	2.2
	Return to practice	50	53.8
	Return to same GP	22	23.7
	Contact OOH service	1	1.1
	Contact emergency services	7	7.5
	Unspecified medical help	3	3.2
	Multiple^a^	2	2.2
Focus of action	No action (conditional warning only)	6	6.5
	Patient (’You come back’)	34	36.6
	Clinician (’I will have another look at it’)	29	31.2
	Both (‘You come back, and I will have another look at it’)	24	25.8
Timescale of action	Not specified	60	64.5
	Named/fixed time (for example, ‘2 weeks’)	28	30.1
	Immediate (for example ,’Go straight to A&E’)	5	5.4
Patient response at the end of the safety-netting advice	No response (including one post-consultation text message where the patient was unable to respond)	9	9.7
	Resistance/misalignment (for example, GP: *‘Any problems let me know’* Patient: *’Yeah, I’ll try and just sort it out myself’, s*ee Supplementary Information S3 for further examples)	24	25.8
	Acknowledgement/acceptance	60	64.5

a These two cases included recontacting the GP practice or the NHS 111 helpline, and recontacting the GP practice or district nursing team. A&E = accident and emergency; OOH = out of hours.

### Delivery of safety-netting advice

When safety-netting advice was given, it was usually verbal (93.1%; *n* = 54/58 consultations, covering 67/72 problems). Four consultations included written advice: in one, the GP texted advice after the call due to a poor telephone line; in two, they sent a text link to a self-help app (known to contain safety-netting advice); and in another, they shared a secondary care letter for one problem and texted a website link for another, both of which contained written safety-netting advice (data not shown).

### Symptoms to look out for

Across 93 safety-netting instances, patients were advised to look out for 182 symptoms/conditionals (Table 5). The mean number per episode was 1.96 (SD 2.03; median = 1; range = 1–13) and per problem 2.60 (SD 2.62; median = 1; range = 1–16, excluding repeats of the same symptom) (data not shown).

**Table 5.** Symptoms to look out for as part of safety-netting advice

**Category**	In all consultations, *n^a^*	Frequency per consultation, ***n* (%)**	
New specific symptom (for example, numbness, breakthrough bleeding, blackout, vomiting, and having problems with passing urine)	83	27 (28.1)	
Current illness/symptoms persist	36	23 (24.0)	
Without a timeframe (‘If it doesn’t work … ’)	12	6 (6.3)	
With timeframe (‘If it persists after 3 months … ’)	24	17 (17.7)	
Other non-specific (for example, ‘develop new symptoms’, ‘if you need’, and ‘not tolerating it’)	18	14 (14.6)	
Worsening/increase in severity of symptoms	14	12 (12.5)	
Return of previous symptoms	9	4 (4.2)	
Concerns/worried/struggling	7	7 (7.3)	
Problems/issues	6	6 (6.3)	
Unwell	4	4 (4.2)	
Haven't heard about an appointment	3	3 (3.1)	
Changes	2	2 (2.1)	

a Includes repeat verbalisations in the same consultation.

Table 5 shows the frequency of symptoms/conditionals for which patients were advised to look out: the most common category was a new specific symptom, which was present in 28.1% (*n* = 27) of 96 consultations, and the second-most common was persistence of current symptoms (24.0%, *n* = 23), of which 73.9% (*n* = 17) included a timeframe.

### Documentation

Of 58 consultations with safety-netting advice, consultation notes were available for 91.4% (*n* = 53). Documentation of advice was found in 64.2% (*n* = 34) of these 53 consultations, covering 58.2% (*n* = 39) of 67 problems. In two cases, no verbal advice was identified, but the documentation could be interpreted as if it had been discussed (data not shown).

### Patient recall of safety-netting advice

Of 96 consultations, 90 patients answered the question: did the HCP say what to do if your problem did not improve or got worse? Among the 55 consultations observed to include safety-netting advice, 69.1% (*n* = 38) of patients correctly recalled receiving it, 14.5% (*n* = 8) did not, 5.5% (*n* = 3) were unsure, and 10.9% (*n* = 6) felt the question did not apply (data not shown).

Of the 35 consultations in which no safety-netting advice was identified, 37.1% (*n* = 13) of patients reported receiving it, 28.6% (*n* = 10) correctly said they had not, 11.4% (*n* = 4) were unsure, and 22.9% (*n* = 8) felt the question did not apply; this gave a total of 62.9% (*n* = 22) non-affirmative responses (data not shown).

### HCPs’ opinions

A total of 28 HCPs completed the clinician pre-study questionnaire; of these, 20 had consultations with patients who had given their consent for their consultation to be used in the study. Supplementary Table S1 summarises both groups’ responses. Most clinicians (*n* = 27, 96.4%) agreed or strongly agreed that safety-netting advice is an important part of their consultations and benefits patient care. Most clinicians (*n* = 26, 92.9%) reported giving verbal safety-netting advice in at least half of their consultations, with 42.9% (*n* = 12) reporting doing so often (50%–69% of the time), 28.6% (*n* = 8) doing so most (70%–89%) of the time, and 21.4% (*n* = 6) doing so almost always (≥90% of the time). Only 7.1% (*n* = 2) reported giving safety-netting advice sometimes (25%–49% of the time). In contrast, written safety-netting advice was used less often: 42.9% (*n* = 12) said they gave it rarely (<25% of the time), 28.6% (*n* = 8) said they gave it sometimes (25%–49% of the time), 7.1% (*n* = 2) often (50%–69% of the time), 10.7% (*n* = 3) most (70%–89%) of the time, and 10.7% (*n* = 3) almost always (≥90%).

The most common reason given for providing safety-netting advice was an equal balance of patient care and medico-legal considerations (53.6%, *n* = 15), followed by ‘mostly patient care, some medico-legal cover’ (39.3%, *n* = 11) (see Supplementary Table S1).

Views were mixed on whether safety-netting advice increases patient demand: 46.4% (*n* = 13) of 28 clinicians disagreed, while 42.9% (*n* = 12) neither agreed nor disagreed (see Supplementary Table S1).

## Discussion

### Summary

This study provides a detailed analysis of safety-netting communication during some GP telephone consultations conducted in seven GP practices in south west England. Safety-netting advice was given in 60.4% of the consultations — which covered fewer than half of problems — and was documented in around two-thirds of these consultations. Advice was predominantly verbal, clinician initiated, and most often delivered during treatment planning or closing phases of consultations. Advice in the treatment planning stages of consultations tended to include specific elements, whereas closing-phase advice was usually generic. Patients were typically advised to seek further help at their GP practice. Safety-netting was more commonly given by younger GPs (aged <45 years). Patient recall was variable, and written advice was rare. Most clinicians regarded safety-netting as important; this was motivated by patient care as well as medico-legal considerations.

### Strengths and limitations

The strengths of this study include the use of real-world data from routinely recorded GP telephone consultations and the fact that, to the authors’ knowledge, this is the first detailed analysis of safety-netting communication given during GP telephone consultations. Broad inclusion criteria captured a wide range of consultation types and presenting problems. Coding was guided by a validated safety-netting tool, with each consultation reviewed at least twice to ensure consistency and reliability. Robust statistical methods, including mixed-effects models, accounted for clustering at the consultation, clinician, and practice levels. Although a Hawthorne effect (clinicians altering their behaviour due to an awareness of being studied) cannot be excluded, clinicians were unaware which patients would participate, so any behaviour change would have needed to be sustained across all telephone consultations during the study period.

Detailed limitations of the archive are reported elsewhere.^19^ Briefly, consultations were unevenly distributed across practices, resulting in an overrepresentation of patients from the least deprived areas. This may limit generalisability, although lower response rates from more-deprived areas are a well-recognised research challenge.^24,25^ In our sample, there were more female than male GPs and patients; however, the GP distribution reflects the proportion of licensed female GPs in the UK (57.7% in 2025).^26^ Consultation numbers by patient gender were consistent with Atherton *et al*’s study^27^ (61.2% female) of 38 714 telephone consultations involving practices in the same geographical location; however, the skew towards White patient participants in our data was greater than in the study by Atherton *et al*;^27^ this may reflect difficulties recruiting a diverse sample, although the small sample size makes this hard to judge.

Some practices also experienced technical issues that prevented extraction of recordings for all 123 patients who consented to join the study, thereby reducing the number available for analysis and limiting statistical power. Finally, the clinical appropriateness of safety-netting advice in each consultation was not assessed.

### Comparison with existing literature

Safety-netting patterns in this study were similar to 318 face-to-face consultations from the original One in a Million study in 2014–2015^14^ (see Supplementary Table S2): rates of safety-netting per consultation (60.4% versus 64.5%) and per problem (43.4% versus 46.3%) were comparable, as were patterns of generic advice in closing phases, specific advice during treatment planning, and advice more often being given for earlier problems and by younger GPs. Documentation was slightly higher in this study (58.2% versus 45.0% per problem), and a greater proportion of instances contained specific advice (64.5% versus 47.2%). This may be related to changes in safety-netting practices over time, which is consistent with out-of-hours data (2013–2020) that showed that increasing documentation and more-specific advice was given in later years.^23^ Additionally, GPs are more likely to document safety-netting when giving specific, rather than generic, advice and in single-problem consultations,^14,22^ both of which were more prevalent in this dataset. Telephone consultations may also offer more flexibility to complete notes during, or after, the call; two-thirds of GPs were audibly typing while consulting^19^ and consultations were shorter than in the original One in a Million archive, potentially allowing more time for documentation if appointment lengths were similar.^19,28^

Compared with other studies, the rate of safety-netting per consultation in this study was similar to 50 telephone recordings from Scottish general practice in 2008 (43%),^17^ but lower than in 53 follow-up consultation recordings collected during 2017 and 2018 (96.2%, *n* = 51/53).^18^

### Implications for research and practice

This study highlights opportunities to strengthen safety-netting in telephone consultations. Although most clinicians recognised its importance for both patient care and medico-legal protection, delivery and documentation were variable. These gaps may pose risks for patients and clinicians, particularly given previous research that has linked absent safety-netting in remote consultations to serious harm.^29^

The study presented here also highlights that GPs usually retain clinical responsibility for patients, with patients not usually advised to seek help outside of their GP surgery. The researchers did not assess the clinical appropriateness of the destinations advised, so the findings describe patterns, rather than judging which option was safer or more efficient. Future work could examine scenario-based clinical consensus on the appropriateness of safety-netting advice and the services to which patients should be directed.

Improving the consistency and clarity of safety-netting communication could enhance patient care and support medico-legal protection in both face-to-face and remote consultations. Technological innovation may help; for example, artificial intelligence (AI) tools could detect when safety-netting advice is absent and prompt clinicians in real time to deliver it. Written after-visit summaries (AVSs) are valued by patients,^30,31^ yet remain rare in the UK compared with the US.^32^ A realist evaluation in UK general practice found that AVSs were most useful when safety-netting advice was important, but were too time consuming for routine use in 2021.^31^ Recent technological advances mean AVSs can be generated automatically using AI scribes, which can also produce patient-friendly documentation, thereby effectively combining the clinician’s notes and an AVS. Finally, further evaluations could test whether generative AI can translate abbreviated GP notes into understandable advice, addressing the finding presented here that around one-third of patients given safety-netting advice did not recall it.

Further research should evaluate the impact of safety-netting advice on patient outcomes, including its role in preventing harm and reducing unnecessary reconsultations, and could also examine the effect of written or multimodal advice on help seeking and safety, as well as how digital technologies might be integrated into remote consulting to support safe, efficient, patient-centred care.

## Open access

This article is Open Access: CC BY 4.0 licence (http://creativecommons.org/licences/by/4.0/).

## Data

Telesafe is a controlled dataset as participants are potentially identifiable by their voices. For more information on accessing the dataset see: https://www.bristol.ac.uk/primaryhealthcare/researchthemes/telesafe
